# Patient-targeted education (ePRO-E) to increase ePRO intent within an Alliance clinical trial (A221805-SI1)

**DOI:** 10.1093/jncics/pkae002

**Published:** 2024-01-16

**Authors:** Ellen M Lavoie Smith, Youmin Cho, Shauna Hillman, Mary R Scott, Elizabeth Harlos, Rachel Wills, Charles Loprinzi, Christina M Wilson, David Zahrieh

**Affiliations:** Department of Acute, Chronic and Continuing Care, University of Alabama at Birmingham School of Nursing, Birmingham, AL, USA; Department of Health Behavior and Biological Sciences, University of Michigan School of Nursing, Ann Arbor, MI, USA; Department of Quantitative Health Sciences, Alliance Statistics and Data Management Center, Mayo Clinic, Rochester, MN, USA; Department of Acute, Chronic and Continuing Care, University of Alabama at Birmingham School of Nursing, Birmingham, AL, USA; Department of Quantitative Health Sciences, Alliance Statistics and Data Management Center, Mayo Clinic, Rochester, MN, USA; Department of Central Protocol Operations, Alliance Protocol Operations Office, Chicago, IL, USA; Department of Medical Oncology, Mayo Clinic, Rochester, MN, USA; Department of Acute, Chronic and Continuing Care, University of Alabama at Birmingham School of Nursing, Birmingham, AL, USA; Department of Quantitative Health Sciences, Alliance Statistics and Data Management Center, Mayo Clinic, Rochester, MN, USA

## Abstract

**Background:**

The Patient Cloud ePRO app was adopted by the National Cancer Institute National Clinical Trials Network (NCTN) to facilitate capturing electronic patient-reported (ePRO) outcome data, but use has been low. The study objectives were to test whether a patient-targeted ePRO educational resource (ePRO-E) would increase ePRO intent (number of users) and improve data quality (high quality: ≥80% of the required surveys submitted) within an ongoing NCTN study.

**Methods:**

The ePRO-E intervention, a patient-targeted educational resource (written material and 6-minute animated YouTube video), was designed to address ePRO barriers. ePRO intent and data quality were compared between 2 groups (*N *=* *69): a historical control group and a prospectively recruited intervention group exposed to ePRO-E. Covariates included technology attitudes, age, sex, education, socioeconomic status, and comorbidity.

**Results:**

Intervention group ePRO intent (78.8%) was statistically significantly higher than historical control group intent (47.1%) (*P *=* *.03). Patients choosing ePRO versus paper surveys had more positive and higher technology attitudes scores (*P *=* *.03). The odds of choosing ePRO were 4.7 times higher (95% Confidence Interval [CI] = 1.2 to 17.8) (*P *=* *.02) among intervention group patients and 5.2 times higher (95% CI = 1.3 to 21.6) (*P = *.02*)* among patients with high technology attitudes scores, after controlling for covariates. However, the 80% submission rate (percentage submitting ≥80% of required surveys) in the ePRO group (30.6%) was statistically significantly lower than in the paper group (57.9%) (*P *=* *.05).

**Conclusions:**

ePRO-E exposure increased ePRO intent. High technology attitudes scores were associated with ePRO selection. Since the ePRO survey submission rate was low, additional strategies are needed to promote high-quality data submission.

The patient is the ultimate authority when assessing the benefits of treatment for controlling symptoms. Patient-reported outcome (PRO) (1) measures can be used to assess patient-perceived benefits and/or to evaluate tradeoffs between quality of life (QOL), toxicity, and survival time. When compared to usual care, research suggests that patients who are routinely monitored using PRO measures may have better QOL and fewer emergency room visits, remain on chemotherapy longer, and also live longer (2).

The US Food and Drug Administration recommends that PROs be collected electronically using cloud-based data capture platforms (3). Based on this recommendation, the Medidata Patient Cloud ePRO app (hereafter referred to as “ePRO app”) has been adopted by the National Cancer Institute (NCI)-supported National Clinical Trials Network (NCTN) (4). The ePRO app can be used to collect survey data directly from patients. Data obtained via the ePRO app are stored within a centralized NCI-supported database—the Medidata Rave Electronic Data Capture (EDC) system—which facilitates rapid access to data and efficient analysis.

Although PROs are typically assessed using paper surveys, electronic data collection (eg, via ePRO) has many advantages: 1) real-time data access, 2) ability to control data collection to occur within a scientifically valid time frame, 3) elimination of data entry by a second person, and 4) decreased risk of data entry errors (5-9). Furthermore, data can be collected via mobile devices, which may enhance patient engagement in PRO collection. Of note, the patient’s email address is not required to use ePRO. However, there are also disadvantages associated with use of electronic data collection tools such as ePRO (eg, limited Internet access in rural areas, less feasible for use by disadvantaged or computer illiterate populations, concerns regarding methods for protecting personal health information).

We define ePRO intent as the patient’s initial interest in using ePRO, which does not assume ongoing compliance. Although ePRO has been employed in several NCTN trials, published data about ePRO intent is limited, as are comparisons of data quality when the same PRO data have been collected via both ePRO and paper surveys. We are aware of data regarding ePRO intent from studies conducted within only two of the six NCTN research groups: NRG Oncology and the Alliance for Clinical Trials in Oncology (Alliance). When given the choice between ePRO and paper surveys, only 37.8% of trial participants chose ePRO, according to pooled data from four NRG Oncology trials (9). Similarly, intent from a recently completed Alliance symptom intervention trial (Alliance A221602) was disappointing: of 690 enrolled patients, only 17% elected to use ePRO (unpublished data). Therefore, additional research is needed to uncover barriers and facilitators to ePRO and critically evaluate whether ePRO is the optimal data collection tool for use within NCTN trials.

Several demographic and clinical factors are associated with the low intent of electronic PRO systems, but most are not modifiable (eg, older age, less education, higher comorbidity, non-White race) (9-11). One of the most basic and modifiable barriers is lack of study participant training and support (12). The ePRO Consortium recommends several educational methods to encourage electronic data collection (12), and one published empirical study supports the scientific premise that education can foster willingness to use electronic PRO systems (6).

The current study addresses a critical gap in scientific knowledge: identifying effective strategies to increase ePRO intent in NCTN clinical trials. The primary objective was to test whether a patient-targeted ePRO-E could increase ePRO intent in an Alliance symptom intervention trial (Alliance A221805). The secondary objective was to compare ePRO versus paper survey data quality (high quality defined as ≥80% submission of all expected surveys). A third objective (exploratory) was to describe predictors of ePRO intent (eg, demographic, clinical, and technology attitudes). Unplanned exploratory analyses examined data submission patterns by 1) chemotherapy treatment cycle, 2) survey, and 3) data submission method (ePRO vs paper surveys) in patients who did and did not have access to ePRO-E.

## Methods

### Design

The ePRO-E sub-study (A221805-SI1) (ClinicalTrials.gov Identifier: NCT04137107) was embedded within the parent study, Alliance A221805: Duloxetine to Prevent Oxaliplatin-Induced Chemotherapy-Induced Peripheral Neuropathy (CIPN): A Randomized, Double-Blind Placebo-Controlled Phase II to Phase III Study. The sub-study was open to enrollment from July 21, 2021 to September 15, 2022. Over the course of Alliance A221805, patients completed PRO surveys at baseline, every 2-3 weeks for 4 to 6 cycles, and 2-4 weeks after the final oxaliplatin treatment.

Alliance A221805-SI1 was activated as part of an amendment (Update 2) to Alliance A221805 13 months after the parent study was initially activated. We compared data between the historical control group (those who were not exposed to ePRO-E prior to Update 2) and a prospectively recruited cohort (experimental group) that was enrolled to Alliance A221805 after Update 2 and were exposed to the ePRO-E intervention. Comparisons to a historical control within the same parent study improved the rigor of the experimental design and provided a practical and defensible way to eliminate the risk of contamination and potential bias introduced by site staff and patients who might share the ePRO-E materials.

### Sample and setting

Historical control and intervention group (ePRO-E) patients were eligible for the sub-study if they were enrolled in a phase II component of Alliance A221805. In addition, ePRO-E intervention participants had to own a smartphone or tablet computer and have access to Wi-Fi. To minimize selection bias, all ePRO-E intervention group participants consented to both the parent and sub-study concurrently (mandatory co-participation). Historical control patients were consented retrospectively after previously consenting to Alliance A221805. The study was approved by the NCI Central Investigational Review Board (IRB), and each accruing site ceded local IRB oversight to the Central IRB. All patients underwent informed consent procedures in accordance with the ethical standards of the Helsinki Declaration and signed IRB-approved consent forms.

### ePRO-E intervention

The ePRO-E resources were developed by Alliance patient advocates, research nurses, clinical research professionals, administrators, ePRO technical experts, and symptom scientists. The team developed simple written instructions (6th-grade reading level) composed of printable materials and a 6-minute animated YouTube video. The video and print materials, packaged in the ePRO-E Toolkit, are generic and applicable to any NCTN trial. The characters depicted in the subtitled video represent diverse people based on race, sex, age, body habitus, clothing and/or cosmetic choices, and disability status (video at https://www.youtube.com/watch?v=LS4lBVyrMsk).

ePRO-E topics were informed by the Unified Theory of Acceptance and Use of Technology (13), published literature (6), and stakeholder input (see Box 1 for included topics). ePRO-E was easily accessible to research staff through the NCI Clinical Trials Support Unit web portal. Patients gained access via email and could download ePRO-E directly to personal electronic devices.

Box 1. ePRO-E educational topics

1. Why use ePRO?
2. Keeping track of your passwords
3. Downloading the app and registration
4. Protecting your security
5. Survey completion
6. Let’s practice
7. Managing ePRO reminder messages
8. Getting technical help

### Outcome measures

ePRO-E intent was assessed based on the number of patients who selected ePRO versus paper surveys before beginning treatment on the parent study. Data submission quality was assessed based on the number of patients who submitted ≥80% of the required surveys at each data collection time point and on the total survey submission rate. The total number of required PRO surveys varied depending on the number of planned chemotherapy treatment cycles (6 or 12). Patients receiving 6 oxaliplatin treatments were required to complete a total of 72 surveys over the primary treatment period, and those receiving 12 treatments would be expected to complete 144 surveys.

Most of the paper and ePRO surveys were completed during routine clinic visits, just before the oxaliplatin infusion. At this infusion visit, patients were reminded to complete one daily survey at home on days 2-6 after every oxaliplatin treatment. The paper user returned these surveys at their next clinic visit, but occasionally, completed paper surveys were returned via mail when circumstances precluded a return clinic visit (eg, illness, travel logistics).

### Covariates

Four surveys were used to collect data on key covariates that could influence technology intent (ie, technology attitudes, education, socioeconomic status, comorbidity) (6,9-12). Survey data were entered into the NCI-supported Medidata Rave EDC for the historical control cohort and collected through Qualtrics for the ePRO-E intervention.

To assess technology attitudes, patients completed the validated adapted Technology Attitudes Survey-A (14). Empirical evidence supports its strong internal consistency reliability (14), and the content validity index (CVI) for the adapted instrument is 0.83 (≥0.8 = excellent content validity) (15). High Technology Attitudes Survey-A scores reflect more positive attitudes toward technology. Since there is no established Technology Attitudes Survey-A cut-point, we defined high scores as those in the top third (>73.3). Participants also answered three additional demographic survey questions about their educational background, annual income, and employment status. Comorbidity was assessed using the Charlson Comorbidity Index (16,17), which quantifies the presence and severity of 19 medical conditions, including diabetes; collapses comorbidities into 4 ordinal categories (0, 1-2, 3-4, and >4); and adjusts for age. A higher score reflects higher comorbidity.

### Historic control group procedures

Consenting historical control patients were only required to complete the demographic and Technology Attitudes Survey-A surveys at a treatment follow-up visit. Study staff completed the Charlson Comorbidity Index based on the comorbid illnesses that were present on the original date of parent study registration. Patients’ preferred data submission methods (paper vs ePRO) had been previously documented.

### Intervention group procedures

Consenting intervention (ePRO-E) group participants were first registered on both the parent and ePRO-E sub-study. Next, participants were emailed a Qualtrics (a web-based survey platform) link to complete the 3 baseline surveys about their education, socioeconomic status, and technology attitudes. After survey completion, the educational video was automatically displayed and a link to the ePRO-E Toolkit was sent via email. Participants were instructed to review the materials, and then choose to submit either electronic or paper surveys before starting the parent study. Study staff recorded patients’ preferences for data submission method, offered additional assistance and/or instructions as needed, and completed the Charlson Comorbidity Index. For paper survey users, surveys were completed onsite at each chemotherapy treatment visit and at home on designated days after chemotherapy. Patients were discouraged from switching between data submission methods. However, because the ePRO app was unavailable for 8 days due to a system outage, one ePRO user submitted paper surveys during this time, and then switched back once the outage was resolved.

### Statistical analyses

Data collection and statistical analysis were conducted by the Alliance Statistics and Data Management Center. Sample size estimates were informed by data from 2 Alliance studies that allowed electronic data capture methods versus paper surveys for data collection. In the absence of ePRO-E, we assumed 15% of patients would choose ePRO over paper surveys. We hypothesized that greater than or equal to 50% of patients who were exposed to ePRO-E would choose ePRO. With a 2-sided Fisher exact test (5% level), we had greater than or equal to 89% power to detect a difference of 50% vs 15% with 40 evaluable patients within each group. Although we planned to enroll 45 patients per group, the historical control group was difficult to accrue during the COVID-19 pandemic, so study enrollment was closed before reaching the accrual target. Despite the small sample size in the accrued control arm (*n *=* *17), the study was able to achieve its primary objective.

Using an intention-to-treat approach, all available data were included in the analysis, regardless of whether the intervention patients viewed ePRO-E. The primary endpoint—whether the patient decided to use ePRO—was a binary outcome (yes, no). For the primary analysis, we compared the ePRO intent rate between the 2 groups by applying the 2-sided Fisher exact test. Logistic regression was used to control for potential confounding factors of gender, age, and technology attitudes. The missing-indicator method (17) was used in the logistic regression model to preserve the sample size used in the primary analysis. We report the unadjusted and adjusted odds ratios (intervention vs historical controls) and corresponding Wald-based 95% confidence intervals. In sensitivity analysis, the primary analysis was repeated in the subset of intervention group patients who indicated that they watched the video. The secondary endpoint was the binary outcome of whether the patient completed greater than or equal to 80% of their expected surveys. The total number of expected surveys was calculated based on the number of treatment cycles completed by each patient. We calculated the proportion of missing surveys over time by group and completion method (ePRO or paper) and the corresponding 95% confidence intervals. *P* values are 2-sided with no adjustment for multiple testing. Descriptive statistics were used for unplanned exploratory analyses. The date of the locked database was February 10, 2023.

## Results

### Demographic and Clinical Characteristics (Table 1)

Seventeen historical control and 52 intervention ePRO-E group patients consented to participate in the sub-study (A221805-SI1) (total *N *=* *69). A main reason why historical control patients were difficult to accrue is that they could not be reached (eg, >18 months since chemotherapy completion, moved, death, no planned follow-up visit due to COVID-19, no response after 3 contact attempts). Most were male (65.2%), White (85.5%), and not Hispanic or Latino (97.1%). The mean participant age was 54.4 years (standard deviation [SD] = 10.3; range 32-78). No significant differences were found between the 2 groups in sex, age, race, ethnicity, chemotherapy regimen (Capecitabine and Oxaliplatin [CAPOX] vs Folic Acid, Fluorouracil and Oxaliplatin [FOLFOX]), highest level of education, comorbidity, or technology attitudes. However, more historical control patients (29.4%) than intervention patients (3.8%) were in the middle-income bracket, and more intervention than control patients preferred not to answer the income question, 48.1% vs 17.6%, respectively. Also, more historical control than intervention patients were currently working (80.0% vs 43.2%).

**Table 1.** Historical control and intervention group demographic and clinical characteristics

	Historical control (*n *=* *17)	ePRO-E intervention (*n *=* *52)	Total (*N *=* *69)	*P*
**Sex**				.57^a^
Male	10 (58.8%)	35 (67.3%)	45 (65.2%)	
Female	7 (41.2%)	17 (32.7%)	24 (34.8%)	
**Age (years)**				.34^b^
*Mean (SD)*	51.6 (9.4)	55.3 (10.5)	54.4 (10.3)	
*Median (Range)*	54.0 (32.0, 65.0)	52.0 (39.0, 78.0)	52.0 (32.0, 78.0)	
**Race**				.24^a^
White	15 (88.2%)	44 (84.6%)	59 (85.5%)	
Asian	0 (0.0%)	4 (7.7%)	4 (5.8%)	
Black or African American	0 (0.0%)	3 (5.8%)	3 (4.3%)	
Not reported: patient refused or not available	1 (5.9%)	1 (1.9%)	2 (2.9%)	
More than one race	1 (5.9%)	0 (0.0%)	1 (1.4%)	
**Ethnicity**				.43^a^
Not Hispanic or Latino	16 (94.1%)	51 (98.1%)	67 (97.1%)	
Hispanic or Latino	1 (5.9%)	1 (1.9%)	2 (2.9%)	
**Chemotherapy regimen**				>.99^a^
CAPOX	11 (64.7%)	34 (65.4%)	45 (65.2%)	
FOLFOX	6 (35.3%)	18 (34.6%)	24 (34.8%)	
**Highest level of education**				.17^a^
At least some graduate school	2 (11.8%)	9 (17.3%)	11 (15.9%)	
At least some college	12 (70.6%)	20 (38.5%)	32 (46.4%)	
At least some high school	1 (5.9%)	7 (13.5%)	8 (11.6%)	
Missing/I prefer not to answer	2 (11.8%)	16 (30.8%)	18 (26.1%)	
**Total combined household income**				**.01** ^a^
$120 000 or above	3 (17.6%)	10 (19.2%)	13 (18.8%)	
$90 000 to $119 999	5 (29.4%)	2 (3.8%)	7 (10.1%)	
Less than $90 000 or Unknown	6 (35.3%)	15 (28.8%)	21 (30.4%)	
Missing/Prefer not to answer/Don't know	3 (17.6%)	25 (48.1%)	28 (40.6%)	
**Employment status**				**.03** ^a^
Currently working	12 (80.0%)	16 (43.2%)	28 (53.8%)	
Not currently working	3 (20.0%)	21 (56.8%)	24 (46.2%)	
Missing	2	15	17	
**Not currently working categories**				>.99^a^
Retired	1 (33.3%)	7 (33.3%)	8 (33.3%)	
On disability	1 (33.3%)	4 (19.0%)	5 (20.8%)	
Not working—other	1 (33.3%)	3 (14.3%)	4 (16.7%)	
On paid sick leave	0 (0.0%)	3 (14.3%)	3 (12.5%)	
Not employed—looking for job	0 (0.0%)	2 (9.5%)	2 (8.3%)	
Not employed—not looking for job	0 (0.0%)	2 (9.5%)	2 (8.3%)	
Missing	14	31	45	
**Charlson comorbidity categories**				.56^a^
0	5 (33.3%)	12 (23.1%)	17 (25.4%)	
1-2	9 (60.0%)	30 (57.7%)	39 (58.2%)	
3-4	1 (6.7%)	10 (19.2%)	11 (16.4%)	
Missing	2	0	2	
**TAS-A** ^c^ **score**				.48^b^
*N*	14	37	51	
*Mean (SD)*	80.0 (11.3)	81.7 (15.3)	81.2 (14.2)	
*Median (range)*	78.7 (65.3, 94.7)	85.0 (40.0, 100.0)	85.0 (40.0, 100.0)	

a Fisher exact *P* value.

b Kruskal-Wallis *P* value.

c TAS-A = Technology Attitude Scale–Adapted.

### ePRO intent (primary outcome)

ePRO intent in those exposed to ePRO-E (78.8%) was statistically significantly higher than in the historical control group (47.1%) (*P *=* *.03). The unadjusted odds ratio (OR) was 4.2 (95% CI = 1.3 to 13.4). This finding was further demonstrated in the logistic regression model adjusting for potential confounding variables (OR 4.7, 95% CI = 1.2 to 17.8; *P* = .02). A sensitivity analysis comparing the historical control group to the intervention group patients who indicated that they watched the video (*n *=* *23) revealed a more marked increase in ePRO intent (91.3%) in the intervention group (*P* = .003).

There were several reasons why study participants did not choose ePRO. Of the 9 patients on the control arm who used paper booklets on cycle 1, 3 (33%) were never offered electronic submission by the study staff. For the 6 patients who used paper booklets despite being offered electronic submission, the primary reasons included “difficulty using smartphone/tablet” (*n *=* *1) and “other, specify” (*n *=* *5) but did not include “data privacy/security concerns,” “no reliable connection/wi-fi,” or “uncomfortable with technology.” For the 12 patients who used paper booklets on the ePRO-E arm on cycle 1, despite being offered electronic submission, the primary reasons given varied and included “data privacy/security concerns” (*n *=* *1), “difficulty using smart phone/tablet” (*n *=* *2), “no reliable connection/wi-fi” (*n *=* *2), “uncomfortable with technology” (*n *=* *2), and “other, specify” (*n *=* *5).

### Data submission quality (secondary outcome)

No statistically significant difference was found between historical control and ePRO-E intervention groups in the percent of patients who submitted at least 80% of the expected surveys (35.3% vs 39.2%, respectively) (Table 2). However, the 80% submission rate for patients who chose to submit their data via ePRO (30.6%) was statistically significantly lower than those who chose paper data submission (57.9%) (*P *=* *.05) (Table 2).

**Table 2.** Frequency of patients completing at least 80% of all expected PRO measures by treatment arm and PRO data collection method

	Treatment Arm			
	Historical Control (*n *=* *17)	ePRO-E (*n *=* *52)	Total (*N *=* *69)	*P*
**Patient completed at least 80% of expected PRO forms**				>.99^a^
No	11 (64.7%)	31 (60.8%)	42 (61.8%)	
Yes	6 (35.3%)	20 (39.2%)	26 (38.2%)	
Missing	0	1	1	

	**PRO Data Collection Method**			
			
	Electronic (*n *=* *49)	Paper (*n *=* *20)	Total (*N *=* *69)	

**Patient completed at least 80% of expected QOL forms**				**.05** ^a^
No	34 (69.4%)	8 (42.1%)	42 (61.8%)	
Yes	15 (30.6%)	11 (57.9%)	26 (38.2%)	
Missing	0	1	1	

a Fisher exact *P* value.

Note: one patient is missing due to no treatment; thus no PRO forms are expected.

### Exploratory outcome predictors

No statistically significant differences were found in demographic or clinical variables between patients who chose ePRO versus paper (Table 3). This includes variables previously thought to matter, such as race, education, income, or comorbidity. However, patients who chose ePRO had a higher mean Technology Attitudes Survey-A score (84.2; SD = 12.8) when compared to the paper survey group (74.7; SD = 15.4) (*P *=* *.03). The odds of choosing electronic data submission (ePRO) were 5.2 times higher (95% CI = 1.3 to 21.6) (*P = *.02*)* among patients with high (>73.3) Technology Attitudes Survey-A scores, after controlling for intervention group, age, and sex.

**Table 3.** Demographic characteristics by data submission method (ePRO vs paper survey)

	Data Collection Method			
	Electronic (*n *=* *49)	Paper (*n *=* *20)	Total (*N *=* *69)	*P*
**Gender**				.78^a^
Male	31 (68.9%)	14 (31.1%)	45 (65.2%)	
Female	18 (75.0%)	6 (25.0%)	24 (34.8%)	
**Age (years, Categorized in Thirds)**				.95^a^
≤50	19 (70.4%)	8 (29.6%)	27 (39.1%)	
50 < age ≤ 61	17 (73.9%)	6 (26.1%)	23 (33.3%)	
<61	13 (68.4%)	6 (31.6%)	19 (27.5%)	
**Age (years, additional category)**				>.99^a^
<65	39 (70.9%)	16 (29.1%)	55 (79.7%)	
≥65	10 (71.4%)	4 (28.6%)	14 (20.3%)	
**Race**				.27^a^
White	39 (66.1%)	20 (33.9%)	59 (89.4%)	
Asian	4 (100.0%)	0 (0.0%)	4 (6.1%)	
Black or African American	3 (100.0%)	0 (0.0%)	3 (4.5%)	
Missing	3	0	3	
**Ethnicity**				>.99^a^
Not Hispanic or Latino	47 (70.1%)	20 (29.9%)	67 (97.1%)	
Hispanic or Latino	2 (100.0%)	0 (0.0%)	2 (2.9%)	
**Chemotherapy Regimen**				>.99^a^
CAPOX	32 (71.1%)	13 (28.9%)	45 (65.2%)	
FOLFOX	17 (70.8%)	7 (29.2%)	24 (34.8%)	
**Highest Level of Education**				.11^a^
At least some graduate school	9 (81.8%)	2 (18.2%)	11 (21.6%)	
At least some college	24 (75.0%)	8 (25.0%)	32 (62.7%)	
At least some high school	3 (37.5%)	5 (62.5%)	8 (15.7%)	
Missing	13	5	18	
**Total Combined Household Income**				.67^a^
$120 000 or above	10 (76.9%)	3 (23.1%)	13 (31.7%)	
$90 000 to $119 999	4 (57.1%)	3 (42.9%)	7 (17.1%)	
Less than $90 000 or Unknown	13 (61.9%)	8 (38.1%)	21 (51.2%)	
Missing	22	6	28	
**Employment Status**				>.99^a^
Currently working	19 (67.9%)	9 (32.1%)	28 (53.8%)	
Not currently working	17 (70.8%)	7 (29.2%)	24 (46.2%)	
Missing	13	4	17	
**Comorbidity Categories**				>.99^a^
0	12 (70.6%)	5 (29.4%)	17 (25.4%)	
1-2	27 (69.2%)	12 (30.8%)	39 (58.2%)	
3-4	8 (72.7%)	3 (27.3%)	11 (16.4%)	
Missing	2	0	2	
**TAS-A** ^c^				**.03** ^b^
N	35	16	51	
Mean (SD)	84.2 (12.8)	74.7 (15.4)	81.2 (14.2)	
Median (range)	86.7 (58.3, 100.0)	72.2 (40.0, 93.3)	85.0 (40.0, 100.0)	

a Fisher exact *P* value.

b Kruskal-Wallis *P* value.

c Technology Attitude Scale–Adapted.

### Unplanned exploratory analyses: Data submission patterns

**Figure 1.**
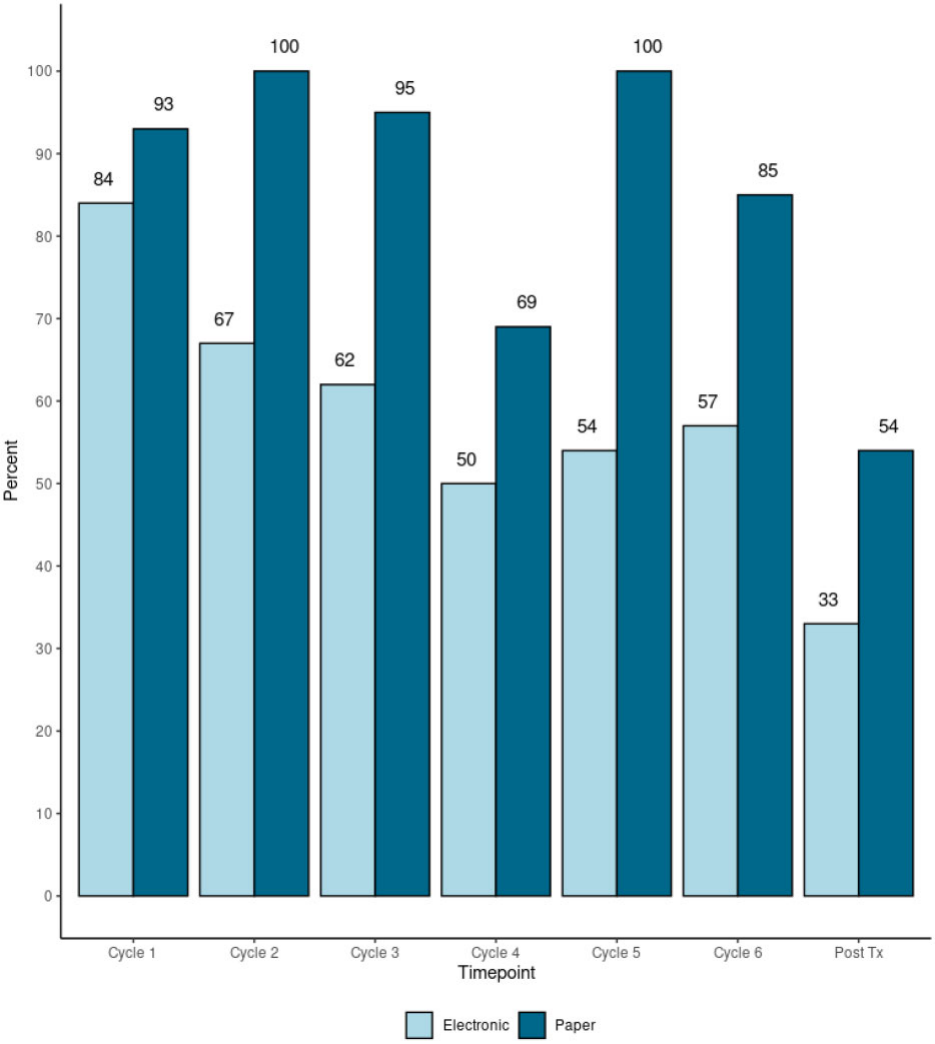
Percent total survey completion by data collection method and chemotherapy treatment time point (cycle).

ePRO survey submission rates (33%-84%) were consistently lower than the paper submission rates across all cycles (Figure 1) and all PRO survey types (Table 4). The total paper survey submission rates by chemotherapy cycle ranged from 54% to 100% (Figure 1). Table 4 illustrates the overall submission rates by chemotherapy treatment time point, PRO survey type, and data submission method. The submission rate for the acute oxaliplatin-induced peripheral neuropathy measure (OIPN) was the lowest at every time point.

**Table 4.** Percent of patients completing PRO surveys by chemotherapy treatment time point (cycle) and data submission method (ePRO vs paper survey)

		PRO Measure					
Data Collection Method	Timepoint	**EORTC** ^a^ **-QLQ-C30** ^b^	**EORTC-QLQ-CIPN20** ^c^	**PRO-CTCAE** ^d^	**BPI-SF** ^e^	**ACUTE OIPN** ^f^	Total
ePRO	Cycle 1	88%	94%	94%	92%	78%	84%
Paper	Cycle 1	100%	100%	95%	100%	89%	93%
ePRO	Cycle 2		72%	72%	72%	65%	67%
Paper	Cycle 2		100%	100%	100%	100%	100%
ePRO	Cycle 3		63%	65%	65%	61%	62%
Paper	Cycle 3		100%	100%	100%	93%	95%
ePRO	Cycle 4		62%	59%	62%	47%	49%
Paper	Cycle 4		93%	93%	93%	61%	68%
ePRO	Cycle 5		62%	62%	62%	59%	60%
Paper	Cycle 5		100%	100%	100%	100%	100%
ePRO	Cycle 6		69%	69%	77%	53%	56%
Paper	Cycle 6		100%	100%	100%	82%	85%

a European Organisation for Research and Treatment of Cancer (EORTC).

b Quality of Life Questionnaire (QLQ-C30).

c Chemotherapy-induced Peripheral Neuropathy (CIPN).

d Common Toxicity Criteria for Adverse Events (CTCAE).

e Brief Pain Inventory Short Form (BPI-SF).

f Oxaliplatin-Induced Peripheral Neuropathy (OIPN).

## Discussion

Lack of patient experience or confidence with using electronic devices and downloading apps, concerns about data security, and lack of access to reliable Internet resources are barriers to electronic data submission (5). The ePRO-E intervention was designed to address most of these barriers, and study findings suggest that it was effective in increasing ePRO intent. The 78.8% ePRO intent rate in the current study is impressively higher than previously documented rates ranging from 17% to 37.8% in other NCTN trials that did not employ methods to encourage ePRO use. Furthermore, given that ePRO-E was created to provide generic, non-protocol-specific information to encourage ePRO use, its use can be expanded more broadly to any study within any NCTN network.

Published studies (9-11) suggest that patients who are older, non-White, and less educated are more likely to prefer paper data submission methods. We did not find evidence that older age predicted paper survey preference. Our study sample did not include an adequate number of non-White participants to confirm or refute that race is a predictor of electronic data submission. However, although not statistically significant, a trend suggested that less educated patients were more likely to select paper surveys. Lastly, our data showed that an individual’s attitudes toward technology is a significant predictor of ePRO intent. This is an encouraging finding because negative attitudes are modifiable targets.

In addition to patients’ attitudes about technology, research professionals’ attitudes may also influence patients’ decisions to use ePRO. Research nurses and clinical research professionals report that electronic data capture systems are not more efficient than paper systems, and patients would rather use paper (9). A mixed-methods study (18) of clinical research professionals provides additional data suggesting that research professionals’ attitudes and beliefs about electronic data collection may sway a patient to select paper over ePRO systems. Future interventions that also target research professionals’ attitudes and beliefs may lead to higher ePRO intent.

Study findings suggest that NCTN trials that rely solely on ePRO data collection methods may be compromised by higher rates of missing surveys, especially when surveys are administered repeatedly and during times when patients are feeling poorly. For example, in our study, in which all participants were receiving oxaliplatin, ePRO acute OIPN survey completion rates were lower than completion rates for all other surveys. Acute OIPN only occurs during days 1-6 after oxaliplatin administration, which is also when patients may be experiencing fatigue and other chemotherapy-related side effects that limit their attention and motivation. Yet, to maintain data validity, acute OIPN survey completion cannot be delayed to when patients feel better. For ensuring that time-sensitive assessment of symptoms such as acute OIPN are completed within a scientifically justified time window, ePRO surveys provide superior data validity than do paper ones: they are available to complete only when symptoms would be present, unlike paper surveys, which can be filled out later. However, a potential downside to ePROs is a higher missed survey completion rate because, if not completed on time, the surveys close permanently. Therefore, the optimal approach to collecting time-sensitive data, especially during times when patients may be less vigilant, may be to combine electronic methods with personal reminders by research staff.

Limitations of the ePRO app system may have contributed to a higher missing survey rate in the ePRO group. Given that the ePRO app directly communicates with patients without study staff support or use of backup methods (eg, email) to remind patients to complete surveys, it is likely that patients could more easily ignore electronic reminders. Furthermore, the ePRO app is designed for smartphones only. Some individuals are less adept at using a smartphone and may have been more compliant if allowed to submit their data via a laptop (eg, larger keyboard and screen, mouse). Further, some individuals may simply just prefer human interaction over technology, and thus were less interested in completing electronic surveys. We also acknowledge that the higher missing survey rate in the ePRO group may have been partly influenced by the complex and burdensome data collection requirements of the parent study. This could be because some ePRO users were confused by the survey response choice options within the app and were making selections independently and with less support from study staff when questions arose. Also, upon closer examination, missing data were missing from people who did not open the email (n = 6) that included the ePRO-E intervention link or who opened the email but did not respond (n = 7). Given that there are many entities competing for attention (eg, marketing promotion emails, social media notifications), it is reasonable to expect that a subset of recipients will not open, read, or respond to an email, and thus will not benefit from an emailed intervention. Given our findings, we conclude that research nurses and clinical research professionals should be aware when ePRO surveys are due, remind patients to complete them, and monitor survey completion within the Medidata Rave EDC system. Clinical research professionals should be trained to closely monitor electronic survey submissions, instead of relying solely on the ePRO app reminders that cue patients to submit their surveys. Perhaps a hybrid approach that combines ePRO data submission methods with monitoring and follow-up by clinical research professionals will result in the highest-quality data, especially with complex data collection schedules. Moreover, because clinical research professionals’ attitudes about technology can also influence patients’ decisions about using ePRO, a similar clinical research professional-targeted ePRO-E training resource should be considered. If well-trained and supportive of electronic data collection methods, clinical research professionals can indirectly influence patients’ willingness and confidence to use electronic data collection systems in the future.

This study has several limitations. The control sample was small, and the target population was homogeneous, including only patients with colorectal cancer who were receiving chemotherapy and a neuropathy intervention; race and ethnic backgrounds were also fairly homogeneous. The COVID-19 pandemic likely contributed to response bias given that historical control patients were quarantined and unable to reconsent to the ePRO-E sub-study at a face-to-face clinic visit. Furthermore, patients and families who had contracted the virus, particularly those from disadvantaged backgrounds, would have been less likely to consent, thereby limiting the representativeness of the historical control cohort. Taken together, these issues limit the generalizability of the findings. Although we found no statistically significant predictors of ePRO intent, the regression analyses were underpowered due to small subgroup sample sizes. Thus, the findings may have been compromised by type II error. Further, research professionals may have discouraged or encouraged ePRO use independent of the ePRO-E intervention.

In conclusion, although educational interventions such as ePRO-E may increase intent of electronic data submission methods, acquisition of the highest-quality data may require more than just a technologic approach. Even in the wake of advanced artificial intelligence and highly sophisticated data collection systems, the best data collection method may still require the human touch.

## Data Availability

Deidentified patient data may be requested from Alliance for Clinical Trials in Oncology via concepts@alliancenctn.org if data are not publicly available. A formal review process includes verifying the availability of data, conducting a review of any existing agreements that may have implications for the project, and ensuring that any transfer is in compliance with the IRB. The investigator will be required to sign a data release form before transfer.
